# The Academic Viewpoint on Patient Data Ownership in the Context of Big Data: Scoping Review

**DOI:** 10.2196/22214

**Published:** 2020-08-18

**Authors:** Martin Mirchev, Iskra Mircheva, Albena Kerekovska

**Affiliations:** 1 Department of Social Medicine and Healthcare Organization Faculty of Public Health Medical University of Varna Varna Bulgaria

**Keywords:** big data, ethics, legal aspects, ownership, patient-generated health data

## Abstract

**Background:**

The ownership of patient information in the context of big data is a relatively new problem, which is not yet fully recognized by the medical academic community. The problem is interdisciplinary, incorporating legal, ethical, medical, and aspects of information and communication technologies, requiring a sophisticated analysis. However, no previous scoping review has mapped existing studies on the subject.

**Objective:**

This study aims to map and assess published studies on patient data ownership in the context of big data as viewed by the academic community.

**Methods:**

A scoping review was conducted based on the 5-stage framework outlined by Arksey and O’Malley and further developed by Levac, Colquhoun, and O’Brien. The organization and reporting of results of the scoping review were conducted according to PRISMA-ScR (Preferred Reporting Items for Systematic Reviews and Meta-Analyses and its extensions for Scoping Reviews). A systematic and comprehensive search of 4 scientific information databases, PubMed, ScienceDirect, Scopus, and Springer, was performed for studies published between January 2000 and October 2019. Two authors independently assessed the eligibility of the studies and the extracted data.

**Results:**

The review included 32 eligible articles authored by academicians that correspond to 3 focus areas: problem (ownership), area (health care), and context (big data). Five major aspects were studied: the scientific area of publications, aspects and academicians’ perception of ownership in the context of big data, proposed solutions, and practical applications for data ownership issues in the context of big data. The aspects in which publications consider ownership of medical data are not clearly distinguished but can be summarized as ethical, legal, political, and managerial. The ownership of patient data is perceived primarily as a challenge fundamental to conducting medical research, including data sales and sharing, and to a lesser degree as a means of control, problem, threat, and opportunity also in view of medical research. Although numerous solutions falling into 3 categories, technology, law, and policy, were proposed, only 3 real applications were discussed.

**Conclusions:**

The issue of ownership of patient information in the context of big data is poorly researched; it is not addressed consistently and in its integrity, and there is no consensus on policy decisions and the necessary legal regulations. Future research should investigate the issue of ownership as a core research question and not as a minor fragment among other topics. More research is needed to increase the body of knowledge regarding the development of adequate policies and relevant legal frameworks in compliance with ethical standards. The combined efforts of multidisciplinary academic teams are needed to overcome existing gaps in the perception of ownership, the aspects of ownership, and the possible solutions to patient data ownership issues in the reality of big data.

## Introduction

### Background

At present, global health systems are undergoing a fundamental change. We have reached the stage of a major transition to ways in which we can improve the generation of and access unimaginable amounts of information. With the emergence and development of big data as a result of the information revolution, we can now manage and transform the approaches by which we control this information and, in health care, as a consequence, the ability to control and cure diseases. On the other hand, the emergence of big data in health care poses additional challenges, especially with regard to the privacy of individuals’ personal data, security, ownership, management, and control.

Personal data, which some call the “21st Century New Oil” [1] or “the new currency,” [2], are generated at an extremely high rate owing to the achievements of modern information and communication technologies (ICTs). In this sense, it is particularly important to address some of the problematic issues directly affecting medical information: Who owns patient information or who would have the fairest claim? Hospitals, researchers, or the patients themselves? Can the information be publicly owned or does it belong to health care providers? Where lies the interest of data carrier developers (software programs, servers, clouds, or social networks)? In general, if patient information is a property of the individuals, will this help improve their health in the reality of big data?

Given the relevance of these issues, and the ensuing additional issues, the lack of proper discussion is more than weird. Isolated debates exist in different places around the world, but interest in improving health care locally, as well as globally, requires concrete and decisive approaches. This suggests going beyond the purely theoretical framework. Considering the importance of this issue, there is a great need to advance our understanding of the relationship between patient information ownership and big data. More importantly, understanding how big data can be used not only to deliver adequate health care and promote traditionally neglected initiatives such as disease prevention and health promotion but also to improve medical research without depriving patients of their potential right to own their medical data.

### Ownership in the Specific Context of Patient Information

In 2009, Hall and Schulman [3] asked, “Who owns medical information? The one who gives care, receives care, or pays for care? All of the above? None of the above? Does it really matter? In the emerging era of electronic health informatics, few other medicolegal questions are more critical, more contested, or more poorly understood.”

Patient information ownership is an issue that requires justifying *self-ownership* [4,5], a modern term of what John Locke considers in 1680 as a *property of his own person* (“every man has a property in his own person: thus nobody has any right to but himself”) [6], that is, patient information or personal health data belong to the characteristics that cannot be separated from the individual [7]. In the 1960s, Westin [8] proposed the idea that personal information should be formally recognized as an object of property rights.

*Ownership* is a complex, often transdisciplinary term that has eroded or has been fragmented depending on the observer’s position and the purpose of the discussion [9]. It is often identified with *privacy*. However, the 2 terms are significantly different. The Oxford Dictionary of Law explains *privacy* as the qualified right to “be free from unwarranted intrusion and to keep certain matters from public view (or surveillance by the state), as recognized in Article 8 of the *European Convention on Human Rights and the *Human Rights Act 1998…*.*” [10]. The term *ownership* is explained in the same dictionary as “the exclusive right to use, possess, and dispose of property, subject only to the rights of persons having a superior interest and to any restrictions on the owner’s rights imposed by agreement with or by act of third parties, or by operation of law” [10]. Therefore, ownership implies certain legal rights over a property along with the explicit right to possess it, such as being able to control, enjoy, use, sell, rent, give away, make profit, or even destroy an item of property. This concept is clear from the perspective of corporeal ownership. It becomes much more complicated when considering incorporeal ownership, such as intellectual property or data and information [11].

Ownership is an important concept because it implies a level of control over the use of personal health data [12]. Other scholars have reviewed the interplay of property law and privacy law on health records and health data, with the bottom line being that neither property nor privacy law is completely applicable to health data or a patient’s ability to control their health data [13].

The scarcity of academic discourse in this field is an interesting phenomenon, given the relevance of the topic [14]. What can be found as research on this subject usually comes from the field of jurisprudence, but there are authors who are known for their works in ethics and health care who also raise this issue [15-17].

Although we are currently witnessing a missing or at least limited academic debate on patient information ownership, the few academic authors who contribute to the debate seem to be grouped around 3 main points of view [14]. According to the first view, this information must be in the public domain [18]. The second view is that patients themselves must be the owners [19]. The third view is that property in itself is not a problem, and the issue can be regulated by other regulations, not specifically by property laws [20,21].

According to Rodwin [18], patient data should be privately owned by patients themselves as a means of protecting their privacy, but there are data that must necessarily be made public to ensure and protect the public interest, including key government initiatives that promote public health, individual patient safety, biomedical research, and not to forget economic interests.

### Big Data in Health Care

Although the use of *huge amount of data* in health care is not a modern phenomenon [22-24], the term *big data* appeared in the 1990s and quickly became popular [25-27]. *Big* is a relative term, especially when it comes to data, and big data usually include data sets that exceed the capabilities of commonly used software tools to store, manage, and process data within a reasonable period of time.

The volume of data is constantly growing, ranging from hundreds of terabytes until a few years ago to thousands of exabytes. Gantz and Reinsel [28] predicted an overall increase in health data by an average of 48% per year. Although there is no precise definition of big data [29], its attributes are well documented in the existing literature. These initially comprised the 3*V* model [30,31]—*Volume, Velocity,* and *Variety— *acknowledged by a number of authors [32,33]. Additional Vs were added by other authors to describe big data [34,35]—*Veracity, Value,* and *Variability* [36-38]. Currently, the term is defined by references from 3 to 15 attributes [39].

Although there are various common definitions of big data [40-42], none of them specifically focus on health, telemedicine, and health care. The European Commission developed the following definition [43] “Big Data in Health refers to large routinely or automatically collected datasets, which are electronically captured and stored. It is reusable in the sense of multipurpose data and comprises the fusion and connection of existing databases for the purpose of improving health and health system performance. It does not refer to data collected for a specific study.” *Yet, not a word about personal patient data and ownership of patient data*.

Over the last few years, methods for aggregating and storing vast amounts of medical data have been improved through various applications [44-46]. Through analyses of big data, diseases can be detected earlier, with better chances and better outcomes for patients [47]. It is now widely believed that evidence-based medicine and, in the long run, personalized and precision medicine must constitute the gold standard of care [48-51]. Technological and informational conditions make ambiguities about the ownership of medical information even more interesting but also unclear. In this regard, the use of big data in health care has yet to prove its importance for clinical support, health insurance, monitoring of diseases and health determinants, treatment optimization, disease prevention and health promotion [52,53], health care improvement in general [54], and medical research, and it also poses serious ethical and regulatory challenges, ranging from risks to individual rights, privacy, and autonomy to transparency of initiatives and trust [55-57].

After the introduction of electronic health records (not to mention the variety of wearables), we are entering a new era of health monitoring. Regardless of its progress, it will be important to determine who will have access to what information, when and how, which ultimately raises the issue of ownership of patient information.

### Considerations for This Study

In this study, we distanced ourselves from publications that refer to “data” as such, without specifying “patient data” explicitly; “property,” argued mainly from the legal point of view as “intellectual property”; and “privacy” that is often identified as “ownership.” It is *ownership* that is within the scope of our research.

Traditionally, *ownership* is debated from the legal standpoint [4,10,26,58-60], yet some authors say that clearly there is no specific data-related legislation that explicitly recognizes ownership in health data in the various EU Member States and in the United States [61-63]. Ultimately, a legal framework reflecting the rights of many stakeholders in the health information market is needed [64,65]. Another line of debate stems from the ethical concerns related to autonomy, privacy, confidentiality, and justice, but barely stressing on ownership as such [66,67].

In this paper, we did not focus on specific legal regulations or ethical considerations, as publications in these fields, to a large extent, do not explicitly address patient data in the context of today’s information reality—big data. It is the perception of the authors that studying legal regulations or ethical considerations requires substantive and more specific research, which is not within the aim and scope of this study.

Our research showed that the peculiarities of *ownership* of patient information in the current data reality have not been the subject of research thus far, and even if they were related, they were not considered as the core issue but as part of studies aimed at something else.

### Choice of Review Type

To map the available evidence in relation to the ownership of patient information in the context of big data, we conducted a scoping review, which by definition best suits the overall objectives of this study to clarify key concepts of an emerging scientific field and identify major sources, gaps, and innovative methods in the available evidence [68]. As we did not find any reviews on the question under investigation, the scoping review is the most appropriate type of review, because it can also be carried out as a stand-alone project, especially when the topic has not yet been extensively reviewed or is of a complex or heterogeneous nature [69-71].

The methodological framework for a scoping review was first outlined by Arksey and O’Malley [72] in 2005. It was further amended by Levac et al [73] in 2010 and by Peters et al [74] in 2015.

Although the terminology and methods for conducting a scoping review are still unclear and not well-described [75], they are constantly improving [76-78], and the use of scoping reviews keeps increasing. The average increase rate of indexed scoping reviews in PubMed for the last 5 years was 51.6% (compared with 16.9% for systematic reviews).

Our choice of scoping review was also determined by the fact that patient information ownership in the context of big data is a relatively new problem and apparently not yet fully recognized by the medical academic community as there are not many publications in this area. In addition, the problem is interdisciplinary, including legal, ethical, medical information, and communication technology aspects, which requires more complex searches and analyses of the available evidence.

## Methods

### Design

We conducted a scoping review, based on the 5-stage framework outlined by Arksey and O’Malley [72], and further developed by Levac et al [73]: (1) identifying the research questions, (2) identifying relevant studies, (3) study selection, (4) data items and data collection process, and (5) collating, summarizing, and reporting results. Specifically, Arksey and O’Malley’s [72] optional sixth stage, *consultations*, was not incorporated in the scoping review, as study quality and evidence strength assessment fall beyond the aims of a scoping review.

The organization and reporting of the results of the scoping review was conducted according to PRISMA-ScR (Preferred Reporting Items for Systematic Reviews and Meta-Analyses and its extensions for Scoping Reviews) [79,80]. As the International Prospective Register of Systematic Reviews does not publish protocols for scoping reviews, the protocol for this scoping review has not been registered or published. A summary of the protocol used in this study is presented in the following subsections.

### Identifying Research Questions

To our knowledge, no scoping review has mapped existing studies on patient information ownership in the context of big data. Therefore, this scoping review aimed to map and assess published studies on this issue. The specific *aim* of this scoping review was to determine how the medical academic community perceives the issue of ownership of patient information in the context of big data, its possible solutions, and implemented practical applications.

To achieve this goal, we identified and summarized 3 main focus areas of the study: (1) *problem*: ownership of patient information, (2) *area*: health care (medicine), and (3) *context*: big data.

On the basis of existing sources and during initial screening of the publications included in the study, the following research questions were defined:

- What is the *scientific area* in which the ownership of patient information in the context of big data is discussed by the academic community?
- What are the *main aspects* of the ownership of patient information in the context of big data as seen by the academic community?
- How does the academic community *perceive* the ownership of patient information in the context of big data?
- Are there any *solutions* for solving the problem of ownership of patient information in the context of big data *proposed* by the academic community? If there are such proposals, what kind of solutions are proposed?
- Are there any *practical applications* for solving the problem of ownership of patient information in the context of big data discussed by the academic community? If there are such applications, what kind of applications?

### Identifying Relevant Studies

Owing to limited access options, we used the following scientific information databases: PubMed, Scopus, Science Direct, and Springer. Given the nature of the topic being investigated, gray literature was not included.

#### Eligibility Criteria

The eligibility criteria for inclusion and exclusion of articles in the scoping review are presented in Table 1.

**Table 1:** Eligibility criteria for inclusion and exclusion of publications in the study.

Criteria	Inclusion criteria	Exclusion criteria
Type of publication, availability	Full-text publication (article, review, commentary, and viewpoint), international research or report, published in a peer-reviewed journal or peer-reviewed congress proceedings	The publication is not in a peer-reviewed journal or peer-reviewed congress proceedings The publication is a study material or a book (book chapter) The publication is not available in full text
Indexing	The publication is indexed in at least one of the scientific information databases under consideration	The publication is not indexed in any of the scientific information databases under consideration
Period	January 2000-October 15, 2019	Before January 2000 and after October 15, 2019
Language	English	All others except English
Focus of the publication	The publication covers all 3 focus areas of the study (problem: ownership of patient information; area: health; and context: big data)	The publication does not cover all 3 focus areas of the study
Relation to research questions	The publication discusses at least one of the research questions (scientific area of publication, aspects of ownership, perception of ownership, proposed solutions, and practical applications)	The publication does not discuss any of the defined research questions The publication discusses mainly human genomics (to our perception, this is a major scientific area that needs to be discussed in a separate study) The publication discusses mainly major and/or specific legal regulations and ethical considerations (to our perception, these are major scientific areas that need to be discussed in separate studies)

#### Search Strategy

An initial standard (title, abstract, and keywords) search in July 2019, using the keywords “ownership,” “health,” and “Big Data,” identifying the 3 focus areas of our study, proved to be inappropriate. To obtain more adequate information, a search strategy was developed. The search strategy was built initially for PubMed using additional keywords such as “owns” and “property” along with “ownership,” and “patient” and “medic*” and “clinic*” along with “health.” It was adopted for the requirements of the different databases. To obtain more relevant and adequate information and to save the screening of thousands, a priori unsuitable for the purposes of our study sources, we used additional filters in compliance with our eligibility criteria and the corresponding databases. The search strategy is described in Multimedia Appendix 1*.*

### Study Selection

On the basis of our eligibility criteria, a systematic search within the chosen bibliographic databases (BDBs) using the conforming search strategy was conducted on October 15, 2019.

Further study selection was performed by assessing the suitability of the identified articles from the initial search to the eligibility criteria. Two authors independently screened the articles based on the eligibility criteria at the title and abstract levels, followed by a full-text screening. Any disagreements between the 2 authors were discussed on a case-by-case basis and resolved in consultation with the third author to ensure consensus. As required, the reference list of all identified articles was searched for additional studies meeting our inclusion criteria. No additional articles were included in our study. Mendeley and Zotero were used to filter duplicated articles and to facilitate the screening process.

### Data Items and Data Collection Process (Charting the Data)

During data extraction, the following article summary information (standard categories) was charted:

- Author
- Title of publication
- Year of publication
- Country of author (in case of several authors, the institution and the country of the corresponding author were considered)
- Authors’ affiliation (academic, research, or nonacademic)
- Type of publication (article or review, congress paper, etc)
- Publication media (journal, conference proceedings, etc)
- Journal title

On the basis of our research questions, we outlined the following *categories for classifying the eligible publications and mapping the available scientific evidence* in our study:

- *Scientific area* of publication in which the ownership of patient information in the context of big data is discussed by the academic community.
- *Aspects of ownership* (of patient information in the context of big data as seen by the academic community)
- *Perception of ownership* (of patient information in the context of big data as seen by the academic community)
- *Proposed solutions* for patient data ownership issues in the context of big data
- *Practical applications* for solving the problem of patient data ownership in the context of big data

All 13 categories were organized in an Excel (Microsoft Corporation) file. Pairs of authors extracted data from the included publications and filled them in an Excel sheet. Any disagreements were resolved by a third author.

As most of the publications eligible for our study were published in impact factor journals and broadly cited, and considering that such bibliometric indicators illustrate a higher quality of the publications, we included 3 additional categories in our study:

- Journal *impact factor*
- *Citation counter* of the publication
- BDB with the largest citation counter of the publication

All publications included in our study were additionally checked in the corresponding information databases and/or the official journal websites to determine the impact factor of the journal and the citation counter of the publications. The data were registered in our Excel file as additional categories (ie, 14-16).

### Collating, Summarizing, and Reporting the Results

All authors discussed and agreed on the registered final results. To analyze the results of our research questions, we used both qualitative and quantitative methods. Most of the results were synthesized in narratives. For numerical summarizations, corresponding to the study categories, we used descriptive statistics (alternative analysis). The majority of the results are displayed in tables, graphs, and narratives.

## Results

### Search Results

A total of 835 publications met our eligibility criteria. Seven publications from other sources, mainly EU and international surveys and reports, were also considered. After filtering the duplicated articles, the total number of potentially appropriate articles was 729.

**Figure 1:**
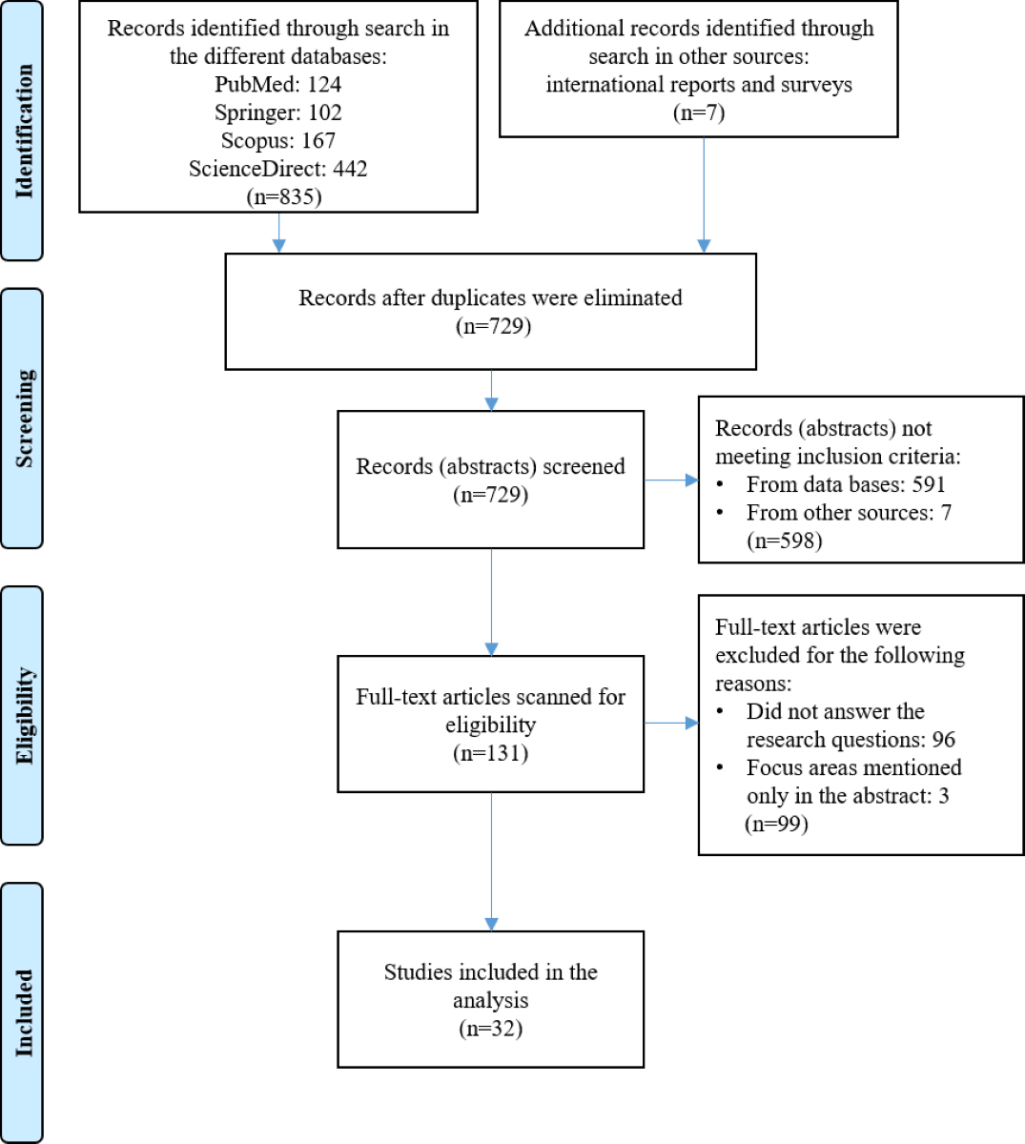
PRISMA (Preferred Reporting Items for Systematic Review and Meta-Analysis) flow diagram of scoping review results.

After screening the file with the bibliographic information and the abstracts of all potentially eligible publications (N=729), manually were excluded duplicated records (n=11), records without an abstract (n=19), records for which no full text was available or full text was not in English (n=85), publications that were not articles or reviews authored by academicians, published in full text in referenced and indexed journals (n=43 plus 7 from other sources), and publications that did not answer any of the research questions (n=433). For a detailed screening, we obtained 131 full-text publications in English. Each of the 131 publications was carefully read and 99 publications were excluded from the study, as they did not cover all 3 focus areas of the study (focus areas mentioned only in the abstract) or did not answer our research questions. The final number of publications included in our study remained to be 32. A PRISMA flow diagram for scoping reviews to visually report the search screening process is presented in Figure 1.

All characteristics of the included publications are presented in Multimedia Appendix 2 [81-112].

### General and Bibliometric Characteristics of the Eligible Publications

Some of the main general and bibliometric characteristics of the sources included in the scoping review are presented in Table 2. The publications are arranged in alphabetical order of the first author’s surname.

**Table 2:** Main general and bibliometric characteristics of the sources included in the scoping review.

References	Year of publication	Countries	Institutions	Types of publication	Journal titles
Andanda [81]	2019	South Africa	Academic	Article	IIC - International Review of Intellectual Property and Competition Law
Andreu-Perez et al [82]	2015	United Kingdom	Academic	Article	IEEE Journal of Biomedical and health informatics
Asche et al [83]	2017	United States	Academic	Article	Pharmacoeconomics
Ballantyne and Stewart [84]	2019	New Zealand	Academic	Article	Asian Bioethics Review
Balthazar et al [85]	2018	United States	Academic	Article	J Am Coll Radiol.
Bietz et al [86]	2018	United States	Academic	Article	J Am Med Inform Assoc
Cvrkel [87]	2019	United States	Academic	Article	Journal of Dentistry
Esmaeilzadeh and Mirzaei [88]	2018	United Kingdom	Academic	Review	J Med Internet Res.
Heitmueller et al [89]	2014	United Kingdom	Academic	Report	Health Affairs
Hölbl et al [90]	2018	Slovenia	Academic	Article	Symmetry
Hulsen [91]	2019	United Kingdom	Academic	Article	Front.Med.
Hunter [92]	2016	United Kingdom	Rethink Technology Research	Article	EMBO Rep.
Ienca et al [93]	2018	Switzerland	Academic	Article	PLoS ONE
Kaplan [94]	2016	United States	Academic	Article	Camb Q Healthc Ethics.
Kaplan [95]	2015	United States	Academic	Article	Camb Q Healthc Ethics.
Kish and Topol [96]	2015	United States	Scripps Research	Extended commentary	Nat Biotechnol.
Kostkova et al [97]	2016	United Kingdom	Academic	Article	Front. Public Health
Kruse et al [98]	2016	United States	Academic	Review	JMIR Med Inform
Kulynych and Greely [99]	2017	United States	Academic	Article	J Law Biosci.
Maher et al [100]	2019	United States	Academic	Article	International Journal of Medical Informatics
Mamoshina et al [101]	2017	United States	Academic	Article	Oncotarget
Mikk et al [102]	2017	United States	MITRE^a^-Research	Viewpoint	JAMA
Mittelstadt and Floridi [103]	2016	United Kingdom	Academic	Review	Sci Eng Ethics
Roehrs et al [104]	2017	Brazil	Academic	Article	J Med Internet Res.
Timmins et al [105]	2018	United Kingdom	Academic	Review	International Journal of Obesity
Vayena and Blasimme [106]	2017	Switzerland	Academic	Article	Journal of Bioethical Inquiry
Vazirani et al [107]	2019	United Kingdom	Academic	Article	J Med Internet Res
Viceconti et al [108]	2015	United Kingdom	Academic	Article	IEEE Journal of Biomedical and health informatics
Willems et al [109]	2019	The Netherlands	Academic	Article	Oral Oncology
Xafis and Labude [110]	2019	Singapore	Academic	Article	Asian Bioethics Review
Yaffe [111]	2019	Canada	Academic	Article	Seminars in Nuclear Medicine
Yue et al [112]	2016	China	Academic	Article	J Med Syst.

^a^MITRE: Massachusetts Institute of Technology Research and Engineering Research.

The 32 publications were from January 2014 to October 2019: 3% (1/32) in 2014 [89]; 13% (4/32) in 2015 [82,95,96,108]; 19% (6/32) in 2016 [92,94,97,98,103,112]; 19% (6/32) in 2017 [83,99,101,102,104,106]; 19% (6/32) in 2018 [85,86,88, 90,93,105]; 28% (9/32) in 2019 [81,84,87,91,100,107,109-111].

The majority of the publications (12/32, 38%) were from the United States [83,85-87,91,94-96,98-102]; 31% (10/32) were from the United Kingdom [82,88,89,92,97,103,105,107,108]. Four (12%) publications were from other European countries (2 from Switzerland [93,106], 1 from Slovenia [90], and 1 from the Netherlands [109]). Six (19%) publications were from other countries: Brazil [104], Canada [111], China [112], New Zealand [84], Singapore [110], and South Africa [81].

The majority of the publications were articles (25/32, 78%). Only 4 (12%) of the publications were reviews [88,98,103,105]. There was also 1 report [89], 1 extended commentary [96], and 1 viewpoint [102].

Almost all of the publications are by authors employed in academic institutions (29/32, 91%). Three (9%) publications [92,96,102] are by academicians employed in research centers.

The majority of the publications are highly cited: 66% (21/32) of publications are cited 10 or more times. The other 10 publications are cited between 1 and 9 times, and only 1 publication from 2019 has not been cited yet.

The publications dealing with the problem of ownership of patient information in the context of big data are published by the academic community in 27 journals covering different scientific areas: (1) medicine, biomedical sciences, and public health—15 journals, 47% (15/32) of publications [83,85, 87,89-93,96,97,101,102,105,109,111]; (2) health and medical informatics—6 journals, 28% (9/32) of publications [82, 86,88,98,100,104,107,108,112]; and (3) medical ethics, law, and health policy—6 journals, 25% (8/32) of publications [81,84,94,95,99,103,106,110].

### Categories for Outlining the Scope of Available Scientific Literature on the Research Questions Under Study

**Figure 2:**
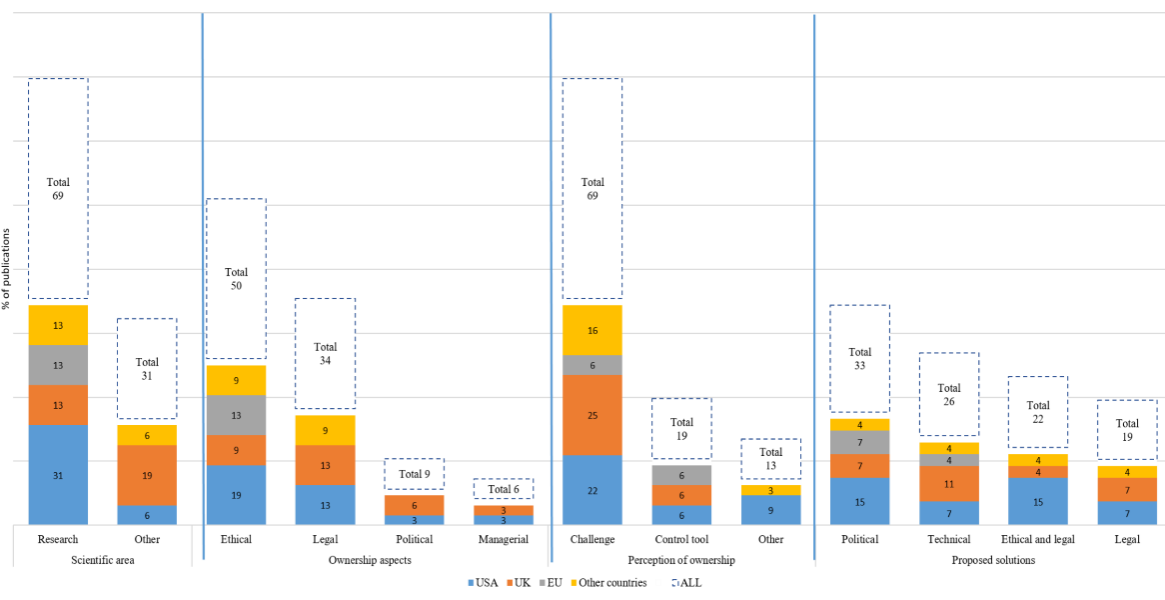
Distribution of the publications by countries and categories, outlining the scope of available scientific literatute on the patient information ownership in the context of big data.

A summarized distribution of the publications included in the scoping review by countries and categories, outlining the scope of the available scientific literature on patient information ownership in the context of big data, is presented in Figure 2.

#### Scientific Areas of the Publications

The distribution of the publications according to the *scientific area* in which the ownership of patient information in the context of big data as discussed by the academic community is presented in Figure 2. The following scientific fields were covered: *Research* (22/32, 69%) [81-86,88,91-95,97,99-101,105,106, 108-111]; *Other* (10/32, 31%), including (1) *ownership and control of medical data* [96,102]; (2) *medical records* [104,107]; (3) *big data use in health care* [89,98]; (4) *application of blockchains* [90,112]; and (5) *ownership as an ethical issue in the context of big data* [87,103].

#### Aspects of Ownership

The distribution of the articles according to the *aspects of ownership* of patient information in the context of big data as seen by the academic community is presented in Figure 2. The following aspects were distinguished: (1) *Ethical* (16/32, 50%) [83,85,87,88,90,93,94,100,102-106,109-111]; (2) *legal* (11/32, 34%), including *legal* [81,82,92,96,101,112] and *legal and ethical* [84,95,99,107,108]; (3) *political* (3/32, 9%)*,* including *political* [89,97] and *political and legal* [86]; and (4) *managerial* (2/32, 6%) [91,98].

#### Perception of Ownership

The distribution of the articles according to the *perception of ownership* of patient information in the context of big data as seen by the academic community is presented in Figure 2. The following alternatives were distinguished: (1) *challenge* (23/32, 72%) [81-89,91-93,97-100,104,105,107-111]; (2) *control tool* (5/32, 16%) [90,101-103,106]; and (3) *other* (4/32, 13%)*,* including: *problem* [95,112], *threat* [94], and *opportunity* [96].

#### Proposed Solutions

The distribution of the articles according to the *proposed solutions* for patient data ownership issues in the context of big data is presented in Figure 2. The following types of solutions were proposed: (1) *political* (9/32, 28%), including *political* [97,98,100,106,108], *political and legal* [86,99], and *political and technical* [109,111]; (2) *technical* (7/32, 22%), including *technical* [82,83,88,90,101,112] and *technical and legal* [107]; (3) *ethical and legal* (6/32, 19%) [85,87,94,95,103,110]; and *legal* (5/32, 16%) [81,89,92,96,102].

Five publications did not propose solutions [84,91,93,104,105].

#### Practical Applications

Only 3 (9%) publications presented *practical applications* for solving the problem of patient data ownership in the context of big data [106,107,112].

## Discussion

### General and Bibliometric Characteristics

The first publication discussing patient information ownership in the context of big data is from 2014, and the number of publications constantly increases. Considering that for the first 10 months of 2019, the number of publications is higher than the average of the previous years, we could expect an increase in the scientific developments in the future.

The authors are mainly from the United States and the United Kingdom. These are the countries where most work was done in the field of medical and health informatics, including the protection of privacy, confidentiality, and ownership of medical data, the use of ICTs, electronic personal records, and big data. However, a new trend was noticed starting in 2018—an increase in scientific developments from authors outside the United States and the United Kingdom, an indicator that academicians from different countries and continents became more involved in discussing and solving the problem of patient information ownership in the context of big data.

Only 3 publications are by academicians not currently employed in academic institutions. The authors are employed in research centers known for their developments in the field of medicine and health, respectively, Rethink Technology Research (UK) and the Scripps Research and the Massachusetts Institute of Technology Research and Engineering, both from the United States, and the publications are published in high impact factor journals.

Most of the publications are articles. The aim of the reviews included in this study is different from the aim of this review. The report, the extended commentary, and the viewpoint included in the study represent important aspects of ownership of patient information and have been repeatedly cited. All publications included in the study are published in scientific peer-reviewed journals with a high impact factor, indexed in one or more BDBs.

We may say that articles dealing with patient information ownership are published predominantly in medical journals (including health and medical informatics). This is reasonable considering that ownership of patient data is discussed mainly as a challenge to medical research, which in the era of big data seems impossible without the involvement of health and medical informatics instruments. Only one-fourth of the articles included in this scoping review are published in specialized journals covering medical ethics, law, and health policy. Hopefully, the publication activity in this area will increase, as medicine and health care, including medical research, are really in urgent need for an adequate legal framework and policies. There are no technical journals, as they do not address the issue of ownership of medical data at all as well as journals that are purely ethical or legal, as they are highly specialized, poorly cited, and in most cases not indexed in the BDBs under consideration.

### Scientific Area of the Publications

Although the issue of ownership of patient information in the context of big data is extremely important, it appears that the academic community’s interest in it is rather low. During PubMed, Scopus, ScienceDirect, and Springer searches, we encountered thousands of publications discussing the application of big data in health care and medicine, hundreds of publications discussing privacy and confidentiality, but only 32 reflecting the issue of ownership of medical data met our inclusion criteria and were included in our study. From the analysis, we identified the following scientific areas in which the publications included in this scoping review can be classified: (1) *research*; (2) *ownership and control of medical data*; (3) *medical records*; (4) *big data use in health care*; (5) *application of blockchains*; and (6) *ownership as an ethical issue in the context of big data*.

The majority of the publications discuss the issue of medical data ownership in the context of big data in the area of *research*: (1) *medical research*: clinical research [82], use of personal health data for medical research [86], precision medicine [91], biomedical research [101,106], obesity research [105], biomedical computing [108], head and neck cancer [109], and medical imaging [111]; (2) *access to and use of health or medical data for research*: impact of data ownership on data sharing and implementation of big data in health-related research [81], validation and data connection [83,85], public-private partnerships to share, analyze, and use biomedical big data [84], the use of personal medical data from wearables [84], data exchange [88], and open data for health care research [97]; (3) *secondary use of medical data for research:* medical data sale [92,94], marketing [95], genetic data from medical records [99], use of passive data [100], and creation and use of data depositories in view of reuse of health data [110]; and (4) *ethical challenges to big data in medical and biomedical research* [93]*.*

Two publications are in the area of *medical records*: ownership discussed as a way of patient communication with personal health records [104] and the use of blockchains for solving medical records problems [107].

Two other publications are in the area of using *big data in health care*: development of public policies to advance the use of big data in health care [89] and the challenges and opportunities of big data in health care [98].

The *application of blockchain* and other ICTs in health care is another research area: research on the use of blockchains in health care [90] and an application using blockchain architecture to assist patients in owning, controlling, and safely exchanging their own data [112].

The ownership of patient information as an *ethical issue in the context of big data* is discussed in 2 publications: data ownership as a complex concept, one of the areas of concern for ethical risk [103] and problems of data access, data ownership, and who has the rightful authority to authorize and profit from the use of the data in mobile health apps [87].

Only 2 of the publications discuss the ownership of medical data as a self-contained study and not as a part of another investigation. Both publications are in the area of *ownership and control of medical data*. The first one discusses the question of why patients should be the owners of their medical data [96], and the other explains why the patients deserve to be the owners and have control over their medical data [102].

Patient data ownership in the context of big data is discussed by the academic community in different scientific fields, each very important and up to date. Although not contradictory, there is a great discrepancy in the publication activity. In addition to the area of research, the other scientific areas are poorly represented and need to be better studied in the future. Future research should also investigate the issue of ownership as a core research question and not as a minor fragment among other topics.

### Aspects of Ownership

The ownership aspects discussed in the publications are not clearly distinguished but can be summarized as follows: (1) *ethical*; (2) *legal*; (3) *political*; and (4) *managerial*, and in combinations such as *legal and ethical* and *political and legal*.

In most publications, ownership of patient data, whether primary or secondary, is considered *ethically, legally or ethically, and legally.* The legal and ethical are not necessarily the same, as Kaplan [95] points out, but their common ground must be found with regard to property.

Data have been created and used since the beginning of civilization [113]. What is changing is the speed at which we create and store data, and the fact that we already have not only methods but also processing capacities that allow us to extract useful information from this vast amount of data [114]. Hence, some of the main *ethical aspects* regarding patient-generated data—who owns it and how it can be used, controlled, exchanged or shared, and preserved—are considered in 50% (16/32) of the publications [83,85,87,88,90,93,94,100, 102-106,109-111], in most cases regarding the use of the data for research.

About one-fifth of the publications consider ownership of medical data in *legal terms.* There are no contradictions among the different opinions. They are predominantly focused on the need for the development of an adequate, alternative, harmonized legal framework, giving the individuals the right to own their health data and the adequate use of that data by the different stakeholders [81,82,92,96,101,112]. Ownership is a concept that is ill-suited for governing rights in big data, and the emergence of big data calls for an alternative normative framework with a view to ensuring fair access while minimizing legal and ethical risks [81]. This assumption is based on the United Nations Educational, Scientific, and Cultural Organization’s (UNESCO) observation that the concept of ownership is no longer an adequate normative framework in the era of big data [115]. According to Hunter [92], as the volume and scope of collected personal health data increases, the greatest requirement is greater transparency regarding the use of that data, which should be harmonized. Kish and Topol [96] state that for the benefits of digital medicine to be fully realized, we not only need to find a shared home for personal health data but also give individuals the right to own them, and the issue of personal identity data is a historical challenge for lawyers. Mamoshina [101] argues that patients have no control over access privileges to their medical records and remain unaware of the true value of the data they have and the idea that they should own it. It cannot be determined whether a proprietary regime that allows total control of the data would actually be the best solution for patients, provided that medical information may be an enigma to them.

The other 5 publications view ownership in *legal and ethical aspects* [84,95,99,107,108]. Ballantyne and Stewart [84] discuss questions of ownership, both of the data and any resulting intellectual property or products. According to Kaplan [95], individuals should be aware of how data about them are collected and used, as using that data might be crucial. Moreover, it is not legally settled whether the data are merely *spoken words* or *property*. Consideration should be given to how these data are used and the ethical development of social norms and laws, as new technologies affect the integrity and protection of health data. On the other hand, according to Viceconti, all medical data are highly sensitive and, in many developed countries, are considered legally owned by the patient, and the health care provider is required to respect patient confidentiality. However, the need for individual confidentiality can be in conflict with the interests of society [108].

Two publications discuss ownership in a *political aspect*. Heitmueller [89] discusses ownership as a policy lever—the devolution of responsibility of data ownership to patients and how patients decide who they want to share their information with can improve health care and may also be a viable alternative to the extremely difficult task of making existing health care systems interoperable. Concerning the opening of patient-generated data to medical research, Kostkova [97] argues that user privacy and ownership of user-generated data remain an underexplored territory from policy and regulatory perspectives while becoming a booming business for the social media industry. In the absence of transparent data ownership regulation, 2 strikingly different approaches emerged for data ownership, usage, and sharing: first, government-regulated clinical and research medical data and, second, private user-generated health data collected from social media, apps, online searches, and wearable devices.

Only one publication presents a survey, carried out among patients and researchers concerning barriers, including political and legal, to the use of personal health data in research, where medical data ownership is discussed in a *political and legal aspect* [86].

To be resolved, the problem of ownership, set initially as ethical, needs the cooperation of legal and political institutions as well as the capabilities of modern technologies. This illustrates a consistent structure from problem to solution—the first step is ethical analysis and the determination of right or wrong, useful or harmful, and fair or unjust; the second step is a subsequent public and political debate; and the third step is a law, regulation, or rulemaking act*.*

Two other publications discuss patient data ownership in a somewhat different aspect, that is, *managerial*. Hulsen [91] states that to fully realize the potential of big data, we must alter the way we work—forming collaborative networks to share samples, data, and methods as well as the legal and ethical frameworks necessary to build and maintain public trust and ensure equitable data access. According to Kruse [98], managerial issues such as governance and data ownership will need to move up on the priority list of organizations, and it should be treated as a primary asset instead of a byproduct of the business. This issue is supported by many authors. In fact, the link between medicine and business, most often expressed in the sale of medical data, both for medical and public health research and research in pharmacoepidemiology as well as for commercial purposes, is a growing trend [92,94,95,97,101].

### Perception of Ownership

The ownership of patient data is considered as a (1) challenge, (2) control tool, (3) problem, and (4) threat and (5) an opportunity. According to our analysis, it is taken primarily as a *challenge.*

The ownership of medical data is generally considered a *challenge to perform medical research* [81,85,92,99], including research in the field of obesity [105], oral oncology, concerning patients having governance of their own data sets, especially when the data are linked to different sources [109], medical imaging [111], the use of passive medical data for research [100], the secondary use of medical data for research, especially in the field of the human genome and electronic medical records [98], and leveraging personal health data for medical research [86].

*Data access challenges*, such as data ownership, data security, and data value, are often considered as barriers to access [83,87], data sharing and exchange [88], access to electronic medical records [107], the use of personal health data [104], and the collection, storage, and reuse of research data [110]. Hulsen et al [91] state that although medical data appear to belong to medical institutions, “the data is the property of the patient and the access and use of that data outside of the clinical realm requires patient consent. This immediately puts a brake on the rapid exploitation of the large volume of data already held in clinical records for precision medicine” [91].

Data ownership, along with data privacy, privacy and security, and data management are serious *social and legal challenges* to big data [82].

In several articles, ownership of medical data is considered a *challenge to health policy* [89], an *ethical challenge* to public-private partnership [84] and modern technologies [108] as well as a *challenge to medical research and business* (sales and data sharing) [97]. An analysis by Ienca et al [93] reveals that the current ethical debate is being largely monopolized by issues of privacy and data protection. However, the issue of data ownership, although distinguished as an ethical challenge, does not actually appear as an ethical priority.

As seen, the challenges, although different in nature, are not contradicting. They concern diverse issues predominantly in performing medical research and activities in association with it as data access, data management, and data sharing. We may argue that these challenges emanate from poor ethical, legal, and policy implications. It will be difficult to overcome such challenges, and the combined efforts and expertise of multidisciplinary research teams are needed.

Apart from being a challenge, ownership of health data is also seen as a *control tool* [102,103,106], controlling access to data [90] and controlling data for biomedical research [101]. There is a slight discrepancy between data access as a challenge and data access as a control tool. This may be resolved if more research is performed.

Two publications consider the ownership of medical data as a *global problem* (along with the use of health data and patient and clinical data protection) for biomedical informatics, patient and physician confidentiality, and regulatory authorities [95] and as a problem (deficiency) for the use of modern blockchain technologies [112].

Kaplan considers the ownership of medical data as a *threat* to the secondary use of data, especially when selling health data [94]. On the contrary, Kish and Topol [96] assume the ownership of medical data as a civil right as an *opportunity* or a strategy for further digitalization of medicine.

The last perceptions of ownership need to be further explored, as it is difficult to judge their significance based on a few opinions.

### Proposed Solutions

A solution to the issue of ownership of patient data in the context of big data is discussed in most publications. Only 5 publications do not offer such a solution [84,91,93,104,105]. Apart from the application of different, mainly new technologies, the solutions are rather proposals to the governments and the governing bodies of the health care institutions and are primarily concerned with enhancing the legal framework, developing adequate policies, finding consensus between ethical and legal aspects, in most cases, mainly related to the right to ownership and control of patients on their own data and the protection of data integrity. Considering that technologies are not a problem, the academic community should be more active in developing concrete solutions and not just proposals, whether technical, legal, or political.

The proposed solutions can be summarized in the following categories: (1) *political;* (2) *technical*; (3) *ethical and legal*; and (4) *legal* and combinations between them: *political and legal*, *political and technical,* and *technical and legal.*

The majority of the proposed solutions concern *policy* decisions, although some of the solutions combine political, legal, or political and technical measures. In general, most of these solutions refer to medical research and the opportunities that big data provide for conducting international medical studies with the exclusive requirement to respect the right of ownership on patient data. Kostkova et al [97] proposed public and political discussions on ownership and responsibility for patient-generated data, as well as the development of a public policy to preserve personal information, which at the same time allows the use of such data to improve public health. According to Kruse et al [98], data ownership and management need to create new business roles that involve analyzing and organizing big data for universal accessibility and sharing and transparency between health care organizations. Maher et al [100] proposed to allow the active involvement of individuals in informed consent procedures and shared ownership of data across countries; researchers should use standardized and validated ways to securely share data, and survey participants should be aware of their data ownership. Extended control through participation management schemes was proposed by Vayena and Blasimme [106] to develop networks of regional cooperatives, potentially worldwide, and to offer open source software for the development of data analysis tools. In this way, “the idea that individuals have direct control over their data can be applied to different national characteristics as well as to international research projects aimed at analyzing data from different countries.” Viceconti et al [108] proposed to fund domain-targeted research that allows specialized solutions to be developed for specific applications in biomedical computing and research.

Two publications combine *policy and law*: developing policies and legal norms for ownership between different countries [86] and offering patients some degree of control over their own data, especially when used for scientific research [99].

*Political and technical solutions* have also been proposed. According to Yaffe [111], instead of severely restrictive policies that do not benefit anyone, reasonable policies regarding the security and privacy of medical data are needed to allow more flexible access and safe exchange (including internationally) of health data between institutions, especially when it comes to medical research and access to various registries (cancer, mortality, rare diseases, etc). Willems et al [109] proposed that instead of bringing together all kinds of data sets in a central comprehensive database, a likely scenario might be that big data users will develop more organic, decentralized virtual networks.

About one-third of the proposed solutions are related to the use of different *technologies*, highlighting solutions related to the application of blockchains: using blockchains to preserve and protect data ownership [88] and to own and share data and health records, and access control [90,112]. The use of blockchains and artificial intelligence enables users to gain ownership and access privileges to their data as well as allow them to sell their data directly to consumers at a fair price [101]. Other technological solutions are also proposed: the use of an identifier for the data collected for the individual with the aim of preserving data security at all levels of the health system, including at every point where the data are collected [82]; the use of distributed networks to provide adequate access to the data, both in efficacy and pharmacoepidemiological studies [83]; and the use of secure multi-party computing—secure multilateral computing that allows third parties to perform calculations with patient data without compromising their integrity [112].

One publication combines *technologies* with *legal* norms. According to Vazirani et al [107], with appropriate regulatory documents and standards, blockchains can serve as a means of managing informed access to health data, as some of their most important features are security, confidentiality, and legal restrictions. This will increase operational interoperability without compromising security while respecting patients’ data confidentiality [107].

With modern technologies, these solutions are fully adequate and feasible. Unfortunately, problems with ownership of medical information in the context of big data cannot have only a technical solution. To provide an adequate solution, it is necessary to develop an appropriate legal framework, backed by appropriate policy decisions and in compliance with the corresponding ethical standards.

There are no proposals for pure ethical solutions, as such solutions cannot be directly applied. They need to be incorporated within the corresponding legal regulations or policies. Six publications propose *ethical and legal* solutions. According to Balthazar et al [85], the community of radiologists, ethics professionals, and computer scientists must negotiate the appropriate way to deal with privacy, confidentiality, data ownership, informed consent, epistemology, and inequalities in the most equitable, ethical way. Cvrkel [87] proposed moving to a consent-focused framework: incorporating data ownership and access and profit agreements into well-developed informed consent. The combined efforts and expertise of lawyers, ethics professionals, and computer scientists on the legal and ethical collection and the use of data, together with the technical knowledge to combine and identify them, can contribute to the development of more informative policies [94,95]. According to Mittelstadt and Floridi [103], taking into account both forms of ownership, the right to *control* and the right to *profit from* data, to exercise adequate data access rights in the big data era, it is necessary to define the terms *commercial* and *scientific* value and to develop specifications for adequate rights and, where necessary, restrictions on access, as well as to modify data protection practices or legislation to oblige *data keepers* to provide data owners with reasonable access to them, so far as this is possible. A similar issue is proposed by Xafis and Labude [110]—state the conditions of access to data repositories—so that data owners can make appropriate decisions regarding the level of access they believe is appropriate for their data and research materials.

One *legal* solution concerns legal arrangements for appropriate *custodianship* of big data to ensure that data subjects maintain some control over access and future uses of their data, while delegating decision making in some matters to the data custodians. Such delegated decision making gives rise to custodial rights, not ownership of the data [81]. This proposal follows UNESCO’s recommendation that a framework with new approaches to ownership and custodianship of personal data should be developed [115]. It may be expected that this idea will contribute to the development of adequate political and legal decisions in view of patient data ownership in the modern big data reality. Other proposals include developing public policy to advance the use of big data in health care, including delegating patient data responsibility to the patients and creating shared networks [89]; improving legal frameworks to protect patient anonymity, informed consent, and data quality assurance [92]; the individuals should be given the right to own their medical data, promoting the ownership of medical data as a civil right and as a major strategy for the further digitalization of medicine, providing new resources to potentially assist any individual who wishes to participate in that [96]; and legal arrangements for ownership as control of patient data (data use agreement) between patients and third parties (data managers, ie, health care and trade organizations), which will allow individuals to control their longitudinal digital records, can improve patient engagement, data accuracy, and health outcomes [102].

### Applications

Despite the many solutions proposed, real applications related to ownership of patient information in the context of big data are only commented on in 3 publications.

One application is presented by Vayena [106]. This is the MIDATA cooperative model developed by MIDATA, Switzerland [116], that provides an example of how individuals can gain control over their own data through new type management mechanisms. The purpose of MIDATA is to store health-related data from a variety of sources and to provide it for scientific projects, while allowing data owners to make their own decisions about their data use. It is a nonprofit organization, but the potential profits generated by consumers will be reinvested in the maintenance of the cooperative or the funding of research [106]. Owners who are registered with MIDATA can actively contribute to medical research and clinical trials by allowing selective access to their own data. They may become members of the cooperative and participate in its management. The MIDATA model is designed for international application: MIDATA Switzerland supports the creation of regional and national MIDATA cooperatives that share the data platform and infrastructure.

The second application is cited by Vazirani et al [107], who described several applications of blockchain technologies for electronic health records, including MedRec [117], which uses blockchain technology and smart contracts to access patient data and manage access permissions. Other applications are mentioned in the review, but they do not affect the ownership of medical data.

The third application is presented by Yue et al [112], who presents an architecture of an application called the Healthcare Data Gateway based on blockchain, which, in addition to patients’ access to their own clinical data and medical records, allows patients to own, control, share, and manage their own data easily and securely without compromising their privacy and provides a potentially new way to improve the health system's intelligence while maintaining patient data ownership. The data are stored in a private blockchain (centralized database with restricted access control, accessible only to authorized or specific users).

### Study Limitations

This study has several major limitations. First, due to limited access options, only 4 scientific BDBs were searched: PubMed, ScienceDirect, Scopus, and Springer. We have not searched other BDBs such as ProQuest, EMBASE (Excerpta Medica dataBASE), and the Web of Knowledge as well as the databases that mainly index technical publications. The search covered the period from January 2000 to October 2019. Thus, the publications that met our inclusion criteria were limited to just 32. Second, our study used only publications in English, which also limited the number of eligible sources. Third, we used mostly sources published in scientific journals authored by academicians. No reports presented at congresses and conferences and published in the corresponding congress proceedings were used, as the full text of the potentially relevant publications was not available even on request. Moreover, reports from different institutions, mainly nongovernmental organizations, could not be used as in most cases they were not authored by academicians and/or were not indexed in the scientific databases.

### Conclusions

Big data in medicine and health care is an issue broadly discussed at present. However, the problem of medical data ownership, especially in the context of big data, no matter its relevance, remains somewhat neglected. What we bear in mind is *ownership* and not *privacy* and *control* that are more broadly represented in the scientific literature by the academic community. The results of the scoping review indicate that the problem of ownership of patient information in the context of big data is poorly researched. Only 2 of the publications discuss the ownership of medical data as a self-contained study and not as a part of another investigation. The problem is not addressed consistently and in its integrity—in terms of ethical, political, and regulatory coherence. The issue of ownership has to be discussed in a more comprehensive way, including ownership problems as a core research question and not just mentioned among other issues.

There are 6 scientific fields in which the publications under review can be classified: research, medical records, use of big data in health care, blockchain applications, ownership and control, and ethics, while the area of research is predominant. The other fields are poorly represented and need to be better studied in the future.

The aspects in which publications consider ownership of medical data are not clearly distinguished, but can be summarized as ethical, legal, political, and managerial, and in combinations such as legal and ethical and political and legal. The largest share is of the publications that consider the ownership of patient information in the context of big data in an ethical aspect. The other aspects need to be further researched.

The ownership of patient data is perceived primarily as a challenge, with this challenge being fundamental to conducting medical research, including access to and use of medical data, which is generally considered a matter of medical ethics. The ownership of medical data is also considered a challenge to policy, ethics, modern technologies, clinical research, and business (data sales and sharing). Apart from being a challenge, ownership of patient data is also seen as a means of control, a problem, a threat, and even an opportunity, also considering primarily medical research. It will be difficult to overcome such challenges, and the combined efforts and expertise of multidisciplinary research teams are needed.

The proposed solutions can be summarized in 3 broad areas: technology, law and ethics, and policy. All the 3 strands are extremely important, but they are not sufficiently represented in the publication activity, and to adequately address the ownership of patient information in the big data information context, these 3 strands need to be combined. To develop and implement an adequate technological solution, it must, in addition to complying with generally accepted ethical standards, comply with certain regulatory documents and policy decisions.

Despite the many solutions proposed, real applications related to the ownership of patient information in the context of big data are commented on in only 3 publications. It is well known that technologies do not prevent the creation of suitable applications. What is missing is adequate policy decisions expressed through the relevant legal frameworks.

The issue of patient information ownership in the context of big data must find its place in the scientific publishing field. It may receive more appropriate answers if special editions of renowned scientific journals are organized to address the issue of ownership of patient information in the context of big data, seminars and roundtables are organized during biomedical forums, and scoping reviews are regularly conducted.

In conclusion, this study may serve as a starting point for future research in this area. It is already evident that technologies are not an obstacle to the development of applications regarding patient data ownership. Cearley et al [118] stated that by 2023, the blockchain will be technically scalable and will support trusted private transactions with the necessary data confidentiality. Given the technological and scientific developments as well as the rapid commercialization of big data in health, the ethical, legal, and policy-making debate on patient data ownership is sure to become more important and widespread.

## Appendix

Multimedia Appendix 1 Search strategy.

Multimedia Appendix 2 Publications included in the scoping review - main characteristics.

## Abbreviations

BDB: bibliographic database
ICT: information and communication technology
PRISMA-ScR: Preferred Reporting Items for Systematic Reviews and Meta-Analyses and its extensions for Scoping Review
UNESCO: United Nations Educational, Scientific, and Cultural Organization
