# Supplementary material for: The Academic Viewpoint on Patient Data Ownership in the Context of Big Data: Scoping Review
Source: J Med Internet Res. 2020 Aug 18;22(8):e22214. doi: 10.2196/22214 (PMC7463395; doi:10.2196/22214)
Supplement: Multimedia Appendix 1 [file jmir_v22i8e22214_app1.docx]

Multimedia appendix 1 - Search strategy

| Database | Search string |
| --- | --- |
| PubMed | (ownership OR owns OR property) AND ("Big data" AND (health* OR patient OR medic* OR clinic*))  Filters applied: *Journal Article, Review, Systematic Review, English, MEDLINE* |
| Science Direct | Find articles with these terms: (ownership OR owns OR property)  In Title, abstract, keyword: ("big data" AND (patient OR health OR medical OR clinical)  Year: 2000 – 2019  Filters applied: *Review articles; Research articles; Conference abstracts* |
| Springer | **With all of the words: (ownership OR owns OR property)**  Where the title contains: "big data" AND (patient OR health OR medical OR clinical) within 2000 - 2019 |
| Scopus | ( ALL ( ownership  OR  owns  OR  property )  AND  TITLE-ABS-KEY ( "Big data"  AND  ( patient  OR  health  OR  medical  OR  clinical ) ) )  AND  ( LIMIT-TO ( SRCTYPE ,  "j" )  OR  LIMIT-TO ( SRCTYPE ,  "p" ) )  AND  ( LIMIT-TO ( SUBJAREA ,  "MEDI" )  OR  LIMIT-TO ( SUBJAREA ,  "SOCI" )  OR  LIMIT-TO ( SUBJAREA ,  "PHAR" )  OR  LIMIT-TO ( SUBJAREA ,  "HEAL" )  OR  LIMIT-TO ( SUBJAREA ,  "PSYC" )  OR  LIMIT-TO ( SUBJAREA ,  "NURS" )  OR  LIMIT-TO ( SUBJAREA ,  "IMMU" )  OR  LIMIT-TO ( SUBJAREA ,  "DENT" ) )  AND  ( LIMIT-TO ( DOCTYPE ,  "ar" )  OR  LIMIT-TO ( DOCTYPE ,  "re" )  OR  LIMIT-TO ( DOCTYPE ,  "cp" ) )  AND  ( LIMIT-TO ( PUBYEAR ,  2019 )  OR  LIMIT-TO ( PUBYEAR ,  2018 )  OR  LIMIT-TO ( PUBYEAR ,  2017 )  OR  LIMIT-TO ( PUBYEAR ,  2016 )  OR  LIMIT-TO ( PUBYEAR ,  2015 )  OR  LIMIT-TO ( PUBYEAR ,  2014 ) )  AND  ( LIMIT-TO ( LANGUAGE ,  "English" ) )  AND  ( LIMIT-TO ( EXACTKEYWORD ,  "Ownership" )  OR  LIMIT-TO ( EXACTKEYWORD ,  "Big Data" ) ) |
